# 2-[(2-Chloro­benzyl­idene)amino]-4,5,6,7-tetra­hydro-1-benzothio­phene-3-carbonitrile

**DOI:** 10.1107/S1600536811032302

**Published:** 2011-08-17

**Authors:** Abdullah M. Asiri, Salman A. Khan, M. Nawaz Tahir

**Affiliations:** a The Center of Excellence for Advanced Materials Research, King Abdulaziz University, Jeddah 21589, PO Box 80203, Saudi Arabia; b Department of Chemistry, Faculty of Science, King Abduaziz University, Jeddah 21589, PO Box 80203, Saudi Arabia; c University of Sargodha, Department of Physics, Sargodha, Pakistan

## Abstract

In the title compound, C_16_H_13_ClN_2_S, the mean planes fitted through all non-H atoms of the heterocyclic five-membered and the benzene rings are oriented at a dihedral angle of 5.19 (7)°. In the crystal, a weak C—H⋯π inter­action occurs, along with weak π–π inter­actions [cenroid–centroid distance = 3.7698 (11) Å].

## Related literature

For information on the use of Schiff bases in pharmaceutical chemistry, see: Lewinski *et al.* (2005 ▶). For related structures, see: Asiri *et al.* (2011**a* ▶,b* ▶).
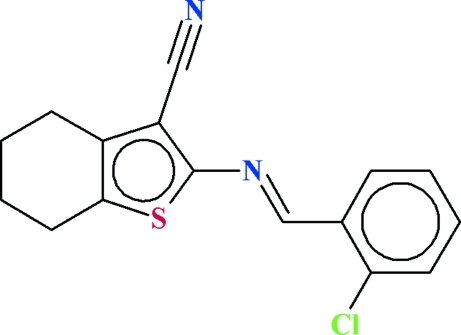


## Experimental

### 

#### Crystal data

C_16_H_13_ClN_2_S
*M* *_r_* = 300.79
Triclinic, 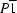

*a* = 8.3383 (4) Å
*b* = 8.6885 (4) Å
*c* = 10.5746 (5) Å
α = 85.975 (2)°
β = 80.806 (2)°
γ = 73.003 (2)°
*V* = 723.00 (6) Å^3^
*Z* = 2
Mo *K*α radiation
μ = 0.40 mm^−1^
*T* = 296 K
0.40 × 0.25 × 0.25 mm

#### Data collection

Bruker Kappa APEXII CCD diffractometer
Absorption correction: multi-scan (*SADABS*; Bruker, 2005 ▶) *T*_min_ = 0.931, *T*_max_ = 0.951
10003 measured reflections
2600 independent reflections
2308 reflections with *I* > 2σ(*I*)
*R*_int_ = 0.019

#### Refinement

*R*[*F* ^2^ > 2σ(*F* ^2^)] = 0.037
*wR*(*F* ^2^) = 0.105
*S* = 1.03
2600 reflections
181 parameters
H-atom parameters constrained
Δρ_max_ = 0.30 e Å^−3^
Δρ_min_ = −0.34 e Å^−3^

### 

Data collection: *APEX2* (Bruker, 2009 ▶); cell refinement: *SAINT* (Bruker, 2009 ▶); data reduction: *SAINT*; program(s) used to solve structure: *SHELXS97* (Sheldrick, 2008 ▶); program(s) used to refine structure: *SHELXL97* (Sheldrick, 2008 ▶); molecular graphics: *ORTEP-3 for Windows* (Farrugia, 1997 ▶) and *PLATON* (Spek, 2009 ▶); software used to prepare material for publication: *WinGX* (Farrugia, 1999 ▶) and *PLATON*.

## Supplementary Material

Crystal structure: contains datablock(s) global, I. DOI: 10.1107/S1600536811032302/nk2106sup1.cif

Structure factors: contains datablock(s) I. DOI: 10.1107/S1600536811032302/nk2106Isup2.hkl

Supplementary material file. DOI: 10.1107/S1600536811032302/nk2106Isup3.cml

Additional supplementary materials:  crystallographic information; 3D view; checkCIF report

## Figures and Tables

**Table 1:** Hydrogen-bond geometry (Å, °) *Cg* is the centroid of the C11–C16 ring.

*D*—H⋯*A*	*D*—H	H⋯*A*	*D*⋯*A*	*D*—H⋯*A*
C5—H5*A*⋯*Cg*^i^	0.97	2.87	3.744 (3)	151

Symmetry code: (i) 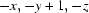.

**Fig. 1.**
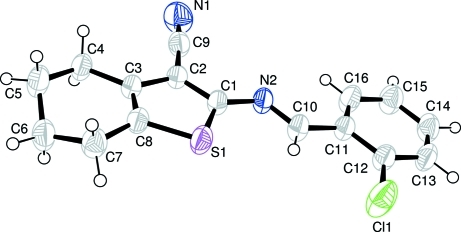
The title compounds with displacement ellipsoids at the 50% probability level. H-atoms are shown as small spheres of arbitrary radii.

**Fig. 2.**
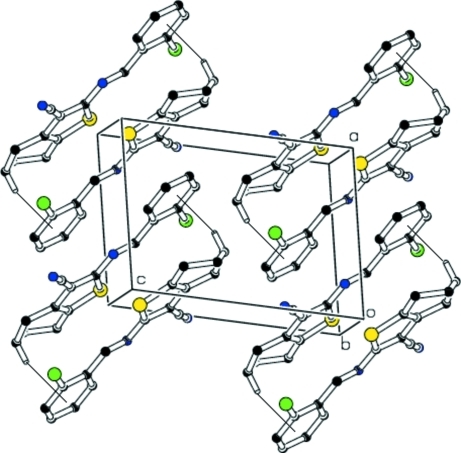
The partial packing (PLATON; Spek, 2009) which shows that C—H···π interactions are present. Only the H-atom involved in π-interaction is present.

## supplementary crystallographic information

### Comment

Schiff-base compounds have been used as intermediate for various reactions (Lewinski, *et al.*, 2005) and medical substrates. The title compound (I), (Fig. 1) has been prepared as a Pharmaceutical intermediate.

We have reported the crystal structure of 2-[(benzo[1,3]dioxol-5-ylmethylene)-amino]-4,5,6,7-tetrahydro-benzo[*b*]thiophene -3-carbonitrile (Asiri *et al.*, 2011*a*) and 2-[(4-Chloro-benzylidene)-amino]-4,5,6,7-tetrahydro-benzo[*b*]thiophene -3-carbonitrile (Asiri *et al.*, 2011*b*) which are related to the title compound.

In (I), the five membered ring A (C1/C2/C3/C8/S1) of 2-amino-4,5,6,7- tetrahydro-1-benzothiophene-3-carbonitrile and the group B (C10–C16/CL1) of 2-chlorobenzaldehyde are planar with r. m. s. deviation of 0.0097 and 0.0020 Å, respectively. The dihedral angle between A/B is 5.19 (7)°. A C—H···π interaction between the six membered rings of 2-amino-4,5,6,7-tetrahydro-1- benzothiophene-3-carbonitrile and the 2-chlorobenzaldehyde group is present (Table 1). π–π interactions [separation: 3.7698 (11) Å] between the heterocyclic five membered and benzene rings are also present.

### Experimental

A mixture of 2-chloro benzaldehyde (0.46 g, 2.4 mmol) and 2-amino-4,5,6,7-tetrahydro-benzo[*b*]thiophene-carbonitrile (0.32 g, 3.3 mmol) in ethanol (15 ml) was heated for 3 h. The progress of the reaction was monitored by TLC. The solid that separated from the cooled mixture was collected and recrystallized from a methanol-chloroform mixture (8:2) to give yellow needles of the title compound (I).

Yellow solid: Yield: 82%, m.p. 450 K.

### Refinement

The H-atoms were positioned geometrically (C–H = 0.93–0.97 Å) and refined as riding with *U*_iso_(H) = x*U*_eq_(C), where x = 1.2 for all H-atoms.

### Crystal data

**Table:**

C_16_H_13_ClN_2_S	*Z* = 2
*M**_r_* = 300.79	*F*(000) = 312
Triclinic, *P*1	*D*_x_ = 1.382 Mg m^−^^3^
Hall symbol: -P 1	Mo *K*α radiation, λ = 0.71073 Å
*a* = 8.3383 (4) Å	Cell parameters from 2308 reflections
*b* = 8.6885 (4) Å	θ = 3.0–25.3°
*c* = 10.5746 (5) Å	µ = 0.40 mm^−^^1^
α = 85.975 (2)°	*T* = 296 K
β = 80.806 (2)°	Rod, yellow
γ = 73.003 (2)°	0.40 × 0.25 × 0.25 mm
*V* = 723.00 (6) Å^3^	

### Data collection

**Table:**

Bruker Kappa APEXII CCD diffractometer	2600 independent reflections
Radiation source: fine-focus sealed tube	2308 reflections with *I* > 2σ(*I*)
graphite	*R*_int_ = 0.019
Detector resolution: 8.20 pixels mm^-1^	θ_max_ = 25.3°, θ_min_ = 3.0°
ω scans	*h* = −9→10
Absorption correction: multi-scan (*SADABS*; Bruker, 2005)	*k* = −10→10
*T*_min_ = 0.931, *T*_max_ = 0.951	*l* = −12→12
10003 measured reflections	

### Refinement

**Table:**

Refinement on *F*^2^	Primary atom site location: structure-invariant direct methods
Least-squares matrix: full	Secondary atom site location: difference Fourier map
*R*[*F*^2^ > 2σ(*F*^2^)] = 0.037	Hydrogen site location: inferred from neighbouring sites
*wR*(*F*^2^) = 0.105	H-atom parameters constrained
*S* = 1.03	*w* = 1/[σ^2^(*F*_o_^2^) + (0.0542*P*)^2^ + 0.253*P*] where *P* = (*F*_o_^2^ + 2*F*_c_^2^)/3
2600 reflections	(Δ/σ)_max_ < 0.001
181 parameters	Δρ_max_ = 0.30 e Å^−^^3^
0 restraints	Δρ_min_ = −0.34 e Å^−^^3^

### Special details

**Table:**

Geometry. Bond distances, angles *etc*. have been calculated using the rounded fractional coordinates. All su's are estimated from the variances of the (full) variance-covariance matrix. The cell e.s.d.'s are taken into account in the estimation of distances, angles and torsion angles
Refinement. Refinement of *F*^2^ against ALL reflections. The weighted *R*-factor *wR* and goodness of fit *S* are based on *F*^2^, conventional *R*-factors *R* are based on *F*, with *F* set to zero for negative *F*^2^. The threshold expression of *F*^2^ > σ(*F*^2^) is used only for calculating *R*-factors(gt) *etc*. and is not relevant to the choice of reflections for refinement. *R*-factors based on *F*^2^ are statistically about twice as large as those based on *F*, and *R*- factors based on ALL data will be even larger.

### Fractional atomic coordinates and isotropic or equivalent isotropic displacement parameters (Å^2^)

**Table:**

	*x*	*y*	*z*	*U*_iso_*/*U*_eq_	
Cl1	0.51410 (9)	0.59840 (7)	−0.31788 (6)	0.0866 (2)	
S1	0.05883 (6)	0.76273 (6)	0.05536 (4)	0.0534 (2)	
N1	0.0435 (3)	0.2524 (2)	0.3132 (2)	0.0764 (7)	
N2	0.24264 (18)	0.44103 (18)	0.02432 (13)	0.0474 (5)	
C1	0.1187 (2)	0.5590 (2)	0.09452 (16)	0.0444 (5)	
C2	0.0275 (2)	0.53040 (19)	0.20896 (15)	0.0409 (5)	
C3	−0.0859 (2)	0.67215 (19)	0.26797 (15)	0.0402 (5)	
C4	−0.1898 (2)	0.6775 (2)	0.39739 (16)	0.0481 (6)	
C5	−0.3072 (3)	0.8451 (2)	0.42743 (19)	0.0596 (6)	
C6	−0.2276 (3)	0.9751 (2)	0.3795 (2)	0.0639 (7)	
C7	−0.1776 (3)	0.9748 (2)	0.23394 (18)	0.0563 (6)	
C8	−0.0812 (2)	0.8060 (2)	0.19563 (16)	0.0450 (5)	
C9	0.0415 (2)	0.3740 (2)	0.26452 (17)	0.0490 (6)	
C10	0.3285 (2)	0.4785 (2)	−0.07655 (17)	0.0515 (6)	
C11	0.4641 (2)	0.3591 (2)	−0.15131 (16)	0.0467 (5)	
C12	0.5574 (2)	0.4006 (2)	−0.26298 (18)	0.0505 (6)	
C13	0.6858 (2)	0.2868 (3)	−0.33329 (18)	0.0553 (6)	
C14	0.7236 (2)	0.1295 (3)	−0.29270 (19)	0.0581 (6)	
C15	0.6354 (3)	0.0838 (3)	−0.1829 (2)	0.0631 (7)	
C16	0.5064 (3)	0.1976 (2)	−0.11318 (19)	0.0580 (6)	
H4A	−0.11493	0.64359	0.46182	0.0577*	
H4B	−0.25684	0.60246	0.40122	0.0577*	
H5A	−0.40913	0.86058	0.38900	0.0715*	
H5B	−0.34027	0.85337	0.51947	0.0715*	
H6A	−0.12747	0.96148	0.41971	0.0766*	
H6B	−0.30654	1.07855	0.40410	0.0766*	
H7A	−0.27819	1.01182	0.19234	0.0676*	
H7B	−0.10754	1.04641	0.20812	0.0676*	
H10	0.30434	0.58581	−0.10391	0.0618*	
H13	0.74600	0.31718	−0.40782	0.0664*	
H14	0.80998	0.05264	−0.33990	0.0697*	
H15	0.66233	−0.02358	−0.15544	0.0757*	
H16	0.44656	0.16560	−0.03915	0.0696*	

### Atomic displacement parameters (Å^2^)

**Table:**

	*U*^11^	*U*^22^	*U*^33^	*U*^12^	*U*^13^	*U*^23^
Cl1	0.0933 (4)	0.0609 (3)	0.0809 (4)	−0.0090 (3)	0.0284 (3)	0.0135 (3)
S1	0.0620 (3)	0.0531 (3)	0.0391 (3)	−0.0172 (2)	0.0119 (2)	−0.0022 (2)
N1	0.0988 (15)	0.0503 (10)	0.0746 (12)	−0.0181 (10)	−0.0035 (11)	0.0008 (9)
N2	0.0421 (8)	0.0579 (9)	0.0393 (8)	−0.0130 (7)	0.0043 (6)	−0.0117 (6)
C1	0.0419 (9)	0.0528 (10)	0.0373 (8)	−0.0138 (7)	0.0020 (7)	−0.0100 (7)
C2	0.0393 (8)	0.0441 (9)	0.0375 (8)	−0.0108 (7)	−0.0007 (7)	−0.0052 (7)
C3	0.0376 (8)	0.0446 (9)	0.0365 (8)	−0.0113 (7)	0.0013 (6)	−0.0051 (7)
C4	0.0494 (10)	0.0483 (10)	0.0400 (9)	−0.0107 (8)	0.0082 (7)	−0.0042 (7)
C5	0.0584 (11)	0.0571 (11)	0.0507 (11)	−0.0076 (9)	0.0146 (9)	−0.0069 (9)
C6	0.0718 (13)	0.0486 (10)	0.0587 (12)	−0.0092 (9)	0.0165 (10)	−0.0123 (9)
C7	0.0635 (12)	0.0425 (10)	0.0542 (11)	−0.0095 (8)	0.0064 (9)	−0.0009 (8)
C8	0.0466 (9)	0.0467 (9)	0.0384 (8)	−0.0130 (7)	0.0044 (7)	−0.0044 (7)
C9	0.0521 (10)	0.0460 (10)	0.0447 (9)	−0.0099 (8)	0.0009 (8)	−0.0091 (8)
C10	0.0462 (10)	0.0592 (11)	0.0445 (10)	−0.0129 (8)	0.0065 (8)	−0.0092 (8)
C11	0.0413 (9)	0.0547 (10)	0.0413 (9)	−0.0130 (8)	0.0040 (7)	−0.0077 (7)
C12	0.0461 (10)	0.0547 (10)	0.0462 (10)	−0.0130 (8)	0.0041 (8)	−0.0027 (8)
C13	0.0453 (10)	0.0701 (12)	0.0447 (10)	−0.0151 (9)	0.0099 (8)	−0.0062 (9)
C14	0.0477 (10)	0.0625 (12)	0.0548 (11)	−0.0054 (9)	0.0064 (8)	−0.0156 (9)
C15	0.0634 (12)	0.0543 (11)	0.0604 (12)	−0.0074 (9)	0.0070 (10)	−0.0042 (9)
C16	0.0578 (11)	0.0596 (12)	0.0486 (10)	−0.0146 (9)	0.0120 (9)	−0.0018 (9)

### Geometric parameters (Å, °)

**Table:**

Cl1—C12	1.7277 (18)	C12—C13	1.381 (3)
S1—C1	1.7322 (17)	C13—C14	1.364 (3)
S1—C8	1.7243 (18)	C14—C15	1.371 (3)
N1—C9	1.139 (2)	C15—C16	1.380 (3)
N2—C1	1.383 (2)	C4—H4A	0.9700
N2—C10	1.265 (2)	C4—H4B	0.9700
C1—C2	1.371 (2)	C5—H5A	0.9700
C2—C3	1.428 (2)	C5—H5B	0.9700
C2—C9	1.422 (2)	C6—H6A	0.9700
C3—C4	1.494 (2)	C6—H6B	0.9700
C3—C8	1.353 (2)	C7—H7A	0.9700
C4—C5	1.521 (2)	C7—H7B	0.9700
C5—C6	1.491 (3)	C10—H10	0.9300
C6—C7	1.529 (3)	C13—H13	0.9300
C7—C8	1.500 (2)	C14—H14	0.9300
C10—C11	1.457 (2)	C15—H15	0.9300
C11—C12	1.393 (3)	C16—H16	0.9300
C11—C16	1.390 (2)		
			
C1—S1—C8	92.06 (8)	C3—C4—H4A	109.00
C1—N2—C10	120.23 (15)	C3—C4—H4B	109.00
S1—C1—N2	126.07 (13)	C5—C4—H4A	109.00
S1—C1—C2	109.88 (12)	C5—C4—H4B	109.00
N2—C1—C2	124.04 (15)	H4A—C4—H4B	108.00
C1—C2—C3	114.07 (14)	C4—C5—H5A	109.00
C1—C2—C9	123.61 (15)	C4—C5—H5B	109.00
C3—C2—C9	122.32 (15)	C6—C5—H5A	109.00
C2—C3—C4	125.31 (14)	C6—C5—H5B	109.00
C2—C3—C8	111.71 (15)	H5A—C5—H5B	108.00
C4—C3—C8	122.86 (15)	C5—C6—H6A	109.00
C3—C4—C5	112.01 (14)	C5—C6—H6B	109.00
C4—C5—C6	112.83 (19)	C7—C6—H6A	109.00
C5—C6—C7	112.49 (17)	C7—C6—H6B	109.00
C6—C7—C8	108.21 (14)	H6A—C6—H6B	108.00
S1—C8—C3	112.24 (13)	C6—C7—H7A	110.00
S1—C8—C7	122.74 (13)	C6—C7—H7B	110.00
C3—C8—C7	124.94 (16)	C8—C7—H7A	110.00
N1—C9—C2	175.9 (2)	C8—C7—H7B	110.00
N2—C10—C11	122.19 (16)	H7A—C7—H7B	108.00
C10—C11—C12	122.04 (15)	N2—C10—H10	119.00
C10—C11—C16	120.95 (16)	C11—C10—H10	119.00
C12—C11—C16	117.01 (16)	C12—C13—H13	120.00
Cl1—C12—C11	120.35 (13)	C14—C13—H13	120.00
Cl1—C12—C13	117.94 (15)	C13—C14—H14	120.00
C11—C12—C13	121.71 (17)	C15—C14—H14	120.00
C12—C13—C14	119.53 (18)	C14—C15—H15	120.00
C13—C14—C15	120.6 (2)	C16—C15—H15	120.00
C14—C15—C16	119.8 (2)	C11—C16—H16	119.00
C11—C16—C15	121.36 (19)	C15—C16—H16	119.00
			
C8—S1—C1—N2	−176.35 (16)	C4—C3—C8—C7	−0.2 (3)
C8—S1—C1—C2	2.04 (14)	C3—C4—C5—C6	−38.3 (2)
C1—S1—C8—C7	175.39 (17)	C4—C5—C6—C7	60.9 (2)
C1—S1—C8—C3	−1.37 (15)	C5—C6—C7—C8	−48.8 (3)
C10—N2—C1—C2	−175.64 (17)	C6—C7—C8—S1	−156.62 (16)
C1—N2—C10—C11	177.94 (16)	C6—C7—C8—C3	19.7 (3)
C10—N2—C1—S1	2.5 (3)	N2—C10—C11—C12	179.42 (17)
S1—C1—C2—C3	−2.3 (2)	N2—C10—C11—C16	−0.9 (3)
S1—C1—C2—C9	176.99 (14)	C10—C11—C12—Cl1	−0.3 (2)
N2—C1—C2—C3	176.19 (16)	C10—C11—C12—C13	179.96 (17)
N2—C1—C2—C9	−4.6 (3)	C16—C11—C12—Cl1	−179.97 (15)
C9—C2—C3—C8	−177.98 (16)	C16—C11—C12—C13	0.3 (3)
C9—C2—C3—C4	5.9 (3)	C10—C11—C16—C15	−179.6 (2)
C1—C2—C3—C4	−174.81 (16)	C12—C11—C16—C15	0.1 (3)
C1—C2—C3—C8	1.3 (2)	Cl1—C12—C13—C14	179.92 (15)
C2—C3—C4—C5	−175.60 (17)	C11—C12—C13—C14	−0.3 (3)
C8—C3—C4—C5	8.7 (2)	C12—C13—C14—C15	−0.1 (3)
C2—C3—C8—S1	0.3 (2)	C13—C14—C15—C16	0.5 (3)
C2—C3—C8—C7	−176.34 (18)	C14—C15—C16—C11	−0.5 (4)
C4—C3—C8—S1	176.53 (13)		

### Hydrogen-bond geometry (Å, °)

**Table:**

Cg is the centroid of the C11–C16 ring.

**Table:**

*D*—H···*A*	*D*—H	H···*A*	*D*···*A*	*D*—H···*A*
C5—H5A···Cg^i^	0.97	2.87	3.744 (3)	151

Symmetry codes: (i) −*x*, −*y*+1, −*z*.
